# Changes in Microbial Safety and Quality of High-Pressure Processed Camel Milk

**DOI:** 10.3390/foods14020320

**Published:** 2025-01-19

**Authors:** Tareq M. Osaili, Dinesh Kumar Dhanasekaran, Fayeza Hasan, Reyad S. Obaid, Anas A. Al-Nabulsi, Amin N. Olaimat, Leila Cheikh Ismail, Nadia Alkalbani, Mutamed Ayyash, Gafar Babatunde Bamigbade, Richard Holley, Adan Shahzadi Cheema, Wael Ahmad Bani Odeh, Khalid Abdulla Mohd, Ayesha Khalid Haji Kamal

**Affiliations:** 1 Department of Clinical Nutrition and Dietetics, College of Health Sciences, University of Sharjah, Sharjah P.O. Box 27272, United Arab Emirates; robaid@sharjah.ac.ae (R.S.O.); lcheikhismail@sharjah.ac.ae (L.C.I.); nalkalbani@sharjah.ac.ae (N.A.); 2 Research Institute of Medical & Health Sciences, University of Sharjah, Sharjah P.O. Box 27272, United Arab Emirates; ddhanasekaran@sharjah.ac.ae (D.K.D.); u00040620@sharjah.ac.ae (F.H.); adan.cheema@uni-bayreuth.de (A.S.C.); 3 Department of Nutrition and Food Technology, Faculty of Agriculture, Jordan University of Science and Technology, P.O. Box 3030, Irbid 22110, Jordan; anas_nabulsi@just.edu.jo; 4 Department of Clinical Nutrition and Dietetics, Faculty of Applied Medical Sciences, The Hashemite University, Zarqa 13133, Jordan; aminolaimat@hu.edu.jo; 5 Department of Women’s and Reproductive Health, University of Oxford, Oxford OX3 9DU, UK; 6 Department of Food, Nutrition and Health, College of Food and Agriculture, United Arab Emirates University (UAEU), Abu Dhabi P.O. Box 15551, United Arab Emirates; mutamed.ayyash@uaeu.ac.ae (M.A.); 202190058@uaeu.ac.ae (G.B.B.); 7 Department of Food Science and Human Nutrition, University of Manitoba, Winnipeg, MB R3T 2N2, Canada; rick_holley@umanitoba.ca; 8 Food Quality and Food Safety Department, University of Bayreuth, 95447 Bayreuth, Germany; 9 Food Studies and Policies Section, Food Safety Department, Dubai Municipality, Dubai P.O. Box 330127, United Arab Emirates; wmodeh@dm.gov.ae (W.A.B.O.); akkamal@dm.gov.ae (A.K.H.K.); 10 Food Inspection Section, Food Safety Department, Dubai Municipality, Dubai P.O. Box 330127, United Arab Emirates; kamohd@dm.gov.ae

**Keywords:** proteins, pathogens, spoilage causing microorganisms, total plate count, lactic acid bacteria, yeasts and molds

## Abstract

High-pressure processing (HPP) is used as a non-thermal approach for controlling microbial viability. The purposes of this study were to (i) establish the decimal reduction times (D-values) for pathogenic bacteria during 350 MPa HPP treatment,; (ii) evaluate the impact of 350 MPa HPP on total plate count (TPC), yeasts and molds (YM), and lactic acid bacteria (LAB) in camel milk; (iii) investigate the behavior of several spoilage-causing bacteria during storage at 4 °C and 10 °C for up to 10 d post-HPP treatment; and (iv) assess the effect of HPP on the protein degradation of camel milk. The D-values for *L. monocytogenes*, *E. coli* O157:H7, and *Salmonella* spp. were 3.77 ± 0.36 min, 1.48 ± 0.08 min, and 2.10 ± 0.13 min, respectively. The HPP treatment decreased pathogenic microorganisms by up to 2 to 3 log cfu/mL (depending on treatment conditions). However, HPP reduced TPC, YM, and LAB by <1 log cfu/mL, regardless of the length of pressure exposure. HPP treatment, even at extended holding times, did not significantly alter either the proteolytic activity or casein micelle structure in camel milk. This study highlights HPP as a promising non-thermal technique for enhancing the microbiological safety of camel milk.

## 1. Introduction

Camel milk has been consumed for centuries in various cultures for its nutritional and medicinal properties. It is opaque white in color, with a faintly sweet odor, though it can sometimes taste salty [1]. Suitable for human consumption as a liquid, camel milk is used to generate various products including cheese, kefir, yogurt, ice cream, and butter [2]. The global camel milk market was estimated to be worth USD 222.4 million in 2022, projected to rise to USD 343.4 million in 2028 [3]. Its utilization is becoming common for its superior nutritional value and low-allergenic nature in contrast to bovine milk [4]. The most common benefits of consuming camel milk are those related to its antibacterial, antiviral, antioxidant, anticancer, and antidiabetic properties. It has been used historically to treat conditions of respiratory, gastric, and intestinal nature [5].

Raw camel milk may harbor microorganisms pathogenic to humans. Common microorganisms responsible for spoilage and potential pathogenicity found in total percentages of contamination associated with raw camel milk include *Staphylococcus aureus* (89.8%), *Streptococcus* spp. (53.7%), *Salmonella* spp. (17.6%), and *E. coli* O157:H7 (31.5%) [6]. A study in Egypt found that the prevalence of *S. enterica* and *E. coli* O157:H7 in camel milk was 5% and 24%, respectively [7,8]. In Algeria, *Listeria* spp. in camel milk was reported to be in 14.3% of the samples [9]. Additionally, the total plate count in camel milk has been recorded as high as 7 log cfu/mL, which significantly detracted from its shelf life [10].

Food quality and safety are critical factors influencing modern consumer choices and are factors which food manufacturers and distributors emphasize. High-pressure processing (HPP) has become increasingly popular as a processing technique which does not use heat and which enhances microbial safety and extends shelf life. HPP involves applying pressure up to 600 MPa commercially, and up to 1000 MPa can be used in laboratory settings, using water as the transmission fluid. Under pressure, time, and temperature conditions normally effective for controlling undesirable microorganisms, HPP treatment has minor effects on nutrient stability. In addition, thermal treatments are known for their negative impact on food nutrients. For instance, HPP preserves vitamin C and ascorbic acid in milk, while pasteurization reduces them by 20 and 16%, respectively [11]. HPP also maintained cytokines in human milk, unlike pasteurization, which lowered levels of interleukin 6, interleukin 8, and TNF alpha [12]. HPP can alter proteins, and this can affect gelation, aggregation, and stability [13]. Notably, proteins of camel milk origin are more vulnerable to denaturation and exhibit lower heat stability and sediment more quickly than bovine milk proteins [14,15]. Another advantage of HPP is that it decreases the need to use chemical preservatives, often seen as undesirable by consumers. It is also an environmentally friendly food preservation technique since it does not produce any waste products like carbon dioxide, excessive heat, etc. [16]. Furthermore, another major advantage, especially in the case of milk, is that HPP can be applied to the product without the need for special containers [16].

On the other hand, one major disadvantage of HPP is that the machine has minimum batch operating requirements to even out the pressure. Secondly, bacterial spores are quite resistant to HPP treatment [17]. While being aware of both the advantages and disadvantages of HPP techniques, as well as their application to various sources as reported in previous studies, our particular interest in exploring this technology is due to its possible benefits outweighing its limitations.

Therefore, to our knowledge, no prior research has examined how HPP treatment influences the microbiological quality of camel milk. Since refrigerators may be frequently opened or malfunction without immediate detection by management, we investigated the growth of microorganisms in camel milk post-HPP treatment under both standard refrigeration conditions (4 °C) and elevated temperatures (10 °C), reflecting potential real-world scenarios. Consequently, this study aimed to explore the effectiveness of HPP treatment at 350 MPa in killing pathogenic bacteria of concern and in controlling spoilage bacteria present in camel milk during 10 d storage at 4 °C or 10 °C. In addition, the study was designed to assess how HPP treatment affected the stability of proteins in camel milk.

## 2. Materials and Methods

### 2.1. Culture Preparation

*L. monocytogenes* (L.M. 7644 and L.M. GLM 5) was acquired from the University of Manitoba in Canada. Similarly, *E. coli* O157:H7 (1934 and 161–84) was procured from the Canadian Science Centre for Human and Animal Health’s National Microbiology Laboratory in Winnipeg, Canada, were utilized in the studies. *Salmonella Typhimurium* 02-8423 and *S. Copenhagen* PT 99 were obtained from Health Canada’s Microbial Hazards Laboratory and the Food Research Institute, Agriculture and Agri-Food Canada, respectively, both in Ottawa, Canada. Before conducting the studies, all strains were stored at −80 °C. Stock cultures of 100 μL of each strain of *L. monocytogenes*, *E. coli* O157:H7 and *Salmonella* spp. were grown in Tryptic Soy Broth (TSB) (Himedia, Maharashtra, India) in tubes and incubated at 37 °C for 24 h. Yeast extract was added at 0.6% to the *L. monocytogenes* tubes as a supplement. Each culture was revived through three consecutive cycles. An initial concentration of 10^8^ cfu/mL was determined by plating. Then, 1 mL of each of *L. monocytogenes*, *E. coli* O157:H7, and *Salmonella* spp. was combined to create a three-strain cocktail combination for each type of bacteria. A total of 6–7 log cfu/mL bacterial population was attained through dilution.

### 2.2. Camel Milk

Camel (*Camelus dromedarius*) milk was sourced from camels at a local farm in the United Arab Emirates (UAE). Milking occurred once daily in the morning. Within 1 h of being obtained, the milk was transported to the laboratory in a refrigerated container filled with crushed ice and the sample temperature was maintained between 2 and 4 °C.

### 2.3. Sample Preparation

In the laboratory, 13 batches, each consisting of 6 × 50 mL lots of camel milk were dispensed in small sterile plastic bottles (Al Bayader International, Sharjah, UAE) made of clear PET material with tamper-proof lids to ensure compatibility with HPP processes. For inoculated samples, freshly prepared *L. monocytogenes*, *E. coli* O157:H7 and *Salmonella* spp. cultures were introduced individually into 50 mL of refrigerated camel milk to reach a population of 6 log cfu/mL (3 batches). After uniformly mixing the cultures through vortexing the packed bottles for 30 s, the samples were located in a container with crushed ice. Similarly, 9 batches of un-inoculated camel milk samples were prepared for background microbiota enumeration (mesophilic total plate count (TPC), lactic acid bacteria (LAB) and yeasts and molds (YM). To prevent leakage, all samples were placed in vacuum pouches, which were subsequently vacuumed sealed using a vacuum-sealing machine (Henkelman B.V., ’s-Hertogenbosch, Netherlands). Finally, all samples were transported to the HPP facility (Smartfood solutions^©^, Dubai, UAE) for HPP treatment. On the same day, raw camel milk was examined to determine the presence of *L. monocytogenes*, *E. coli* O157:H7 and *Salmonella* spp. [18,19,20]) and found to be negative.

### 2.4. Treatment

Camel milk samples were treated in HPP equipment operated at 10 °C (Hyperbaric, Model 55, Burgos, Spain). The treatment involved applying a pressure of 350 MPa for 1, 2, 3, 4, or 5 min for samples inoculated with *L. monocytogenes* strains and *Salmonella* spp. However, the samples inoculated with *E. coli* O157:H7 were treated at 300 MPa for 1–5 min. All analyses were conducted in triplicate on different days. Several preliminary experiments were conducted to assess the extent of microorganism reduction using different pressure levels (250, 300, 350, 400, and 450 MPa). At pressure levels higher than those chosen, the reductions were remarkably high with all pathogenic microorganisms tested, resulting in the rapid elimination of bacteria within a few seconds.

After the treatment, un-inoculated and inoculated camel milk samples were kept at refrigerated (4 °C) and abusive temperatures (10 °C) for 0, 1, 4, 7, and 10 d. Samples that were stored at 4 °C and 10 °C for up to 10 d were used to assess the stability and quality of camel milk.

### 2.5. Microbial Enumeration

Following the HPP treatment (0, 1, 4, 7, and 10 d), 10 mL samples were placed in a level II biosafety cabinet, aseptically pipetted into bottles containing 90 mL of sterile peptone water (Himedia, Maharashtra, India) and mixed thoroughly for 2 min. Thereafter, serial dilutions were prepared and plated on selective agars in duplicate. The selective agars used for the tests were as follows: Listeria Selective Agar with the Modified Listeria Selective Supplement (Himedia, Maharashtra, India) for *L. monocytogenes*, Sorbitol MacConkey Agar with the Cefixime-Tellurite Supplement (Himedia, Maharashtra, India) for *E. coli* O157:H7 and Xylose Lysine Deoxycholate Agar (Himedia, Maharashtra, India) for *Salmonella* spp. An overlay of 10 mL Tryptone Soy Agar (Himedia, Maharashtra, India) was used to coat selective agars and resuscitate injured cells [21]. Optimum growth conditions were provided by aerobically incubating the plates at 37 °C for 48 h.

For the enumeration of mesophilic TPC and LAB, 1 mL from an appropriate dilution was plated on Plate Count Agar (Himedia, Maharashtra, India) and Man Rogosa Sharpe Agar (Himedia, Maharashtra, India) using the pour plate technique followed by incubation at 30 °C for 3 d. LAB plates were incubated under anaerobic conditions (CO_2_ incubator, Binder GmbH, Tuttlingen, Germany). Similarly, the pour plate technique was also used for YM (Sabouraud Dextrose Agar, Himedia, Maharashtra, India) followed by incubation at 25 °C for 5 d.

### 2.6. Calculation of D-Values

The log_10_ cfu, which represents organisms that survived the HPP treatment, was plotted against the recorded exposure time to ascertain the decimal reduction time (D-value). The data points were correlated via linear regression. The D-value was then derived by calculating the reciprocal of the slope of the regression line.

The D-values for pathogens in camel milk were calculated using [22]

D-value = [t_2_ − t_1_]/[log A − log B]

where A and B denote the number of survivors after HPP treatment for times t_1_ (0 min) and t_2_ (5 min), respectively.

### 2.7. Sodium Dodecyl-Sulfate Polyacrylamide Gel Electrophoresis (SDS-PAGE)

The protein components of the camel milk samples (1 batch) were analyzed post HPP treatment using the reducing SDS-PAGE technique with slight modification [15]. Briefly, the hand-cast 1 mm thick polyacrylamide gels were prepared with 12% and 4% resolving and stacking gels, respectively. Sample preparation was performed by mixing 1 mL camel milk with 9 mL of deionized water, and then 35 µL of the mixture was added to 65 µL of freshly prepared 4 X Lamelli sample buffer solution containing 50 mM dithiothreitol (DTT) as a reducing agent. The mixture was then heated for 5 min at 90 °C and 5 µL of the mixture was loaded on a pre-cast polyacrylamide gel. A pre-stained BLUeye protein ladder (Sigma-Aldrich, St. Louis, MO, USA) was used as a marker. The electrophoresis of the SDS-PAGE gels was conducted at 200 V using Mini-PROTEAN^®^ Tetra vertical electrophoresis cell system (Bio-Rad Laboratories Inc., Hercules, CA, USA) filled with 1 X running buffer solution. The gel was then stained for 1 h using SDS-PAGE stain solution with constant shaking, treated with destaining I solution for 45 min, and destaining solution II overnight. The resulting gels were washed with deionized water, and gel images were captured (Gel Doc XR+, Bio-Rad Laboratories Inc., Hercules, CA, USA) against a UV/White light conversion screen.

### 2.8. Statistical Analysis

Linear regression analyses were performed for plots of survivors versus time for the pathogens using Microsoft Excel, while GraphPad Prism Version 7.0 (Graph Pad Software, Inc., La Jolla, CA, USA) generated the corresponding graphical representation. The effect of treatment and their interaction effect on the microbial populations (TPC, YM and LAB) in camel milk was confirmed by a two-way analysis of variance (ANOVA) with subsequent post hoc analysis by Tukey’s HSD using the IBM SPSS Statistics software package (version 26, Chicago, IL, USA). The effect of storage temperatures on the microbial populations (TPC, YM and LAB) was quantified through two-tailed, unpaired student *t*-tests using GraphPad Prism Version 7.0 (H.J. Motulsky, Prism 7 Statistics Guide, GraphPad Software Inc., San Diego, CA, USA). Each test series and measurements in the entire experiment were replicated at three different time intervals on different days. All statistical analyses were considered significant at *p* < 0.05.

## 3. Results and Discussion

### 3.1. Effect of HPP Activity on Food Borne Pathogenic Bacteria

Commercial applications of HPP may entail the use of pressures as high as 600 MPa, although laboratory settings using smaller HPP vessels may safely achieve pressures as high as 1000 MPa [23]. However, based on our pilot study on camel milk inoculated with *L. monocytogenes*, *E. coli* O157:H7, and *Salmonella* spp. cultures, viable microbes were destroyed after short exposures to pressures of 400 MPa. Therefore, 350 MPa HPP pressure was chosen for this study except for *E. coli* O157:H7 as it was even more sensitive to HPP. Consequently, *E. coli* O157:H7 challenge tests in camel milk were conducted at 300 MPa.

**Figure 1:**
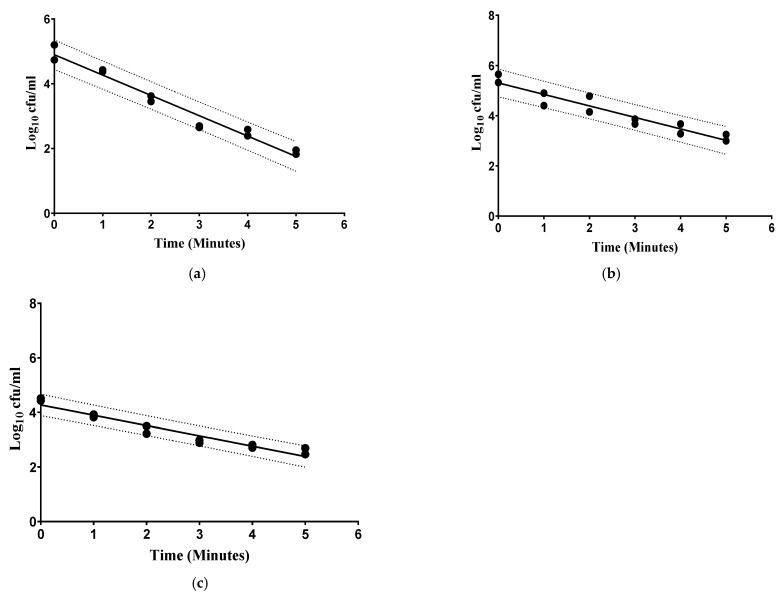
Survival curve of (**a**) *Salmonella* spp. (R^2^ = 0.960, correlation coefficient = −0.586), (**b**) *L. monocytogenes* (R^2^ = 0.928, correlation coefficient = −0.402) with confidence and predication bands (dotted lines in camel milk samples) at 350 MPa, and (**c**) *E. coli* O157:H7 spp. (R^2^ = 0.938, correlation coefficient = −0.433) at 300 MPa.

Figure 1 shows the non-thermal HPP destruction curves of *L. monocytogenes*, *E. coli* O157:H7, and *Salmonella* spp. No shoulders or irregularities in the curves were observed. The regression curves of all the pathogens had a determination coefficient > 0.90 suggesting a strong alignment fit between the data and the model.

In this study, the D-value for *L. monocytogenes* was 3.77 ± 0.36 min. This is surprisingly higher when compared to a previous study where *L. monocytogenes* in Cheddar-type raw milk cheese treated at 300 MPa had a D-value of 3.6 min [24]. Meanwhile, in low-salt white brined cheese, a D-value of 0.52 min at 600 MPa was observed [25]. The D-value of *L. monocytogenes* at 600 MPa was 2.43 min in raw bovine milk and 1.52 min in orange juice, respectively. Meanwhile, it was 0.87 min for peach juice and 1.63 min for brain heart infusion broth, respectively. This suggests the influence of various food matrices on the D-value [26]. Besides the food matrix, the low value in the brined cheese as compared to the other liquid foods could have been influenced by the different water activity (aw) of the foods tested. Although a higher aw in orange/peach juices and milk provides a better environment for the bacteria to proliferate than does cheese, lower aw can also shield microorganisms from the destructive effects of HPP [27]. Differences in strain susceptibility may also influence outcomes.

The D-value of *E. coli* O157:H7 was 1.48 ± 0.08 min. In comparison, *E. coli* O157:H7 in Cheddar-type raw cheese had D-values of 4.4 min at a pressure of 300 MPa [24]. On the other hand, in low-salt, white brined cheese, a D-value of 1.13 min at 600 MPa was observed [24]. Meanwhile, in apple juice, *E. coli* (strain ATTCC 28055) showed D-values of 9.22 (250 MPa), 2.95 (300 MPa), and 0.80 min (350 MPa), respectively [28].

Lastly, in this study, the D-value of *Salmonella* spp. was 2.10 ± 0.13 min. No previous studies in the literature have investigated the effect of HPP on *Salmonella* spp. in liquid camel milk. A reduction of 6 log cfu/mL was reported for *Salmonella* spp. in carrot juice after approximately 4 min of treatment at 300 MPa [29]. The differences in D-values could attributed to various factors, including microbial strain, its response and adaptation to the surrounding medium, pressure level, pressure-holding time, and the type and composition of the food matrix [30].

As expected, the D-value of *L. monocytogenes* was higher than that of *Salmonella* spp. and *E. coli* O157:H7 in camel milk. This is due to studies having shown that Gram-positive bacteria exhibit greater resistance to HPP than Gram-negative bacteria [31,32]. Gram-positive microorganisms have a thicker cell wall mainly composed of peptidoglycan, which comprises 90% of the cell wall compared to the 5–10% peptidoglycan content in the Gram-negative cell wall [33]. HPP treatment decreased pathogenic microorganisms by up to 2 to 3 log cfu/mL (depending on treatment conditions ≤ 5 min at 350 MPa). A similar effect was expected with spoilage-causing microorganisms, namely TPC, LAB, and YM. However, HPP reduced these microorganisms only by < 1 log cfu/mL regardless of exposure time (Table 1, Table 2 and Table 3).

### 3.2. Effect of HPP Activity on Spoilage Causing Bacteria

The initial TPC load was 4.3 and 4.1 log cfu/mL at 4 and 10 °C, respectively. This decreased to a minimum of 3.5 log cfu/mL (after 1 day of storage after 5 min HPP treatment) and 3.4 log cfu/mL (without storage/day 0 after 2 min HPP treatment), respectively. Similarly, the initial YM load was 4.2 and 4.5 log cfu/mL at 4 and 10 °C, respectively. This decreased to a minimum of 3.3 log cfu/mL (after 1 day storage post 5 min of HPP treatment) and 3.3 log cfu/mL (without storage post 5 min of HPP treatment), respectively. On the other hand, in the case of LAB, the initial load was 3.9 and 3.5 log cfu/mL at 4 and 10 °C, respectively. This decreased to a minimum of 3.0 log cfu/mL (after 4-day storage post 5 min of HPP treatment) and 3.4 log cfu/mL (without storage post 1 min of HPP treatment), respectively.

Increasing the time of HPP treatment did not have any significant effect (*p* > 0.05) in decreasing any of the three microbial populations monitored (TPC, LAB, YM). This could have been because the surviving microorganisms were largely Gram-positive bacteria (i.e., LAB) or were spore formers which are more resistant to HPP treatment. As per one study, LAB exhibit pressure resistance by becoming more curved and small, and by increasing the thickness of their cell wall [34]. Pressure-resistant LAB are capable of maintaining their cell integrity and a uniform protoplasm with smaller cavities (due to the pressure), and they have a higher glucose utilization capacity (by almost 9.6%) in comparison to pressure sensitive strains. Through these changes, LAB could increase the pressure resistance in the strain by up to 1222 times. The LAB in our study may have undergone these changes to resist the increasing time of HPP treatment, although this would need further investigation. With respect to YM, interestingly, moderately high pressures (50 to 300 MPa) can encourage spore germination [35]. These pressures activate the nutrient receptors of the spore. In our study, a partial destruction by HPP or the above explanation of activation of spores could be one reason why the decrease in microbial numbers, which was expected, was not observed. However, this could perhaps be dealt with by using another mild hybrid technology to destroy the spores (after a short period of storage) because a spore that is at stage II of germination is more vulnerable to destruction as compared to a spore that is at stage I of germination, majorly due to the higher content of water at the core [35]. In another study, it was observed that HPP altered the membrane permeability of spores causing the leakage of intra cellular contents to the outside environment. However, in comparison to a thermal treatment, this effect was milder in an HPP treatment [36]. Furthermore, no significant (*p* > 0.05) beneficial effect was observed in terms of decreasing microbial numbers by keeping the milk under 4 °C post-HPP treatment. However, storing the milk under 4 °C was needed to prevent an increase in TPC, YM, and LAB. All three groups proliferated post-HPP treatment at 10 °C. Under refrigeration conditions, at the end of the storage period (10 d), HPP treated milk samples were lower in TPC, YM, and LAB by up to 0.6, 1.2, and 0.6 log cfu/mL, respectively, as compared to non-HPP treated samples. Meanwhile, the increase in TPC and YM after storage at 10 °C, despite HPP treatment, was 1.2 log cfu/mL (after 1 min HPP treatment and storage for 10 d), and with LAB it was 2.0 log cfu/mL (after 3 min of HPP treatment and storage for 10 d). This clearly indicates that microorganisms present were not eliminated by the HPP treatment and were injured. Microorganisms under stress conditions develop various mechanisms of survival like synthesis of protective proteins, outer membrane vesicles, and protective biofilms [37]. Once microorganisms find themselves in more favorable environmental conditions, they proliferate, which in this study was at 10 °C.

The higher levels of LAB in HPP-treated samples compared to the control after 10 days of storage at 10 °C can be attributed to the resistance of LAB to HPP. As Gram-positive bacteria, LAB have structural characteristics that confer higher resilience to pressure compared to Gram-negative microorganisms. HPP likely eliminated more sensitive microorganisms (e.g., Gram-negative bacteria) in the milk, reducing microbial competition and allowing LAB to thrive in the post-treatment environment.

**Figure 2:**
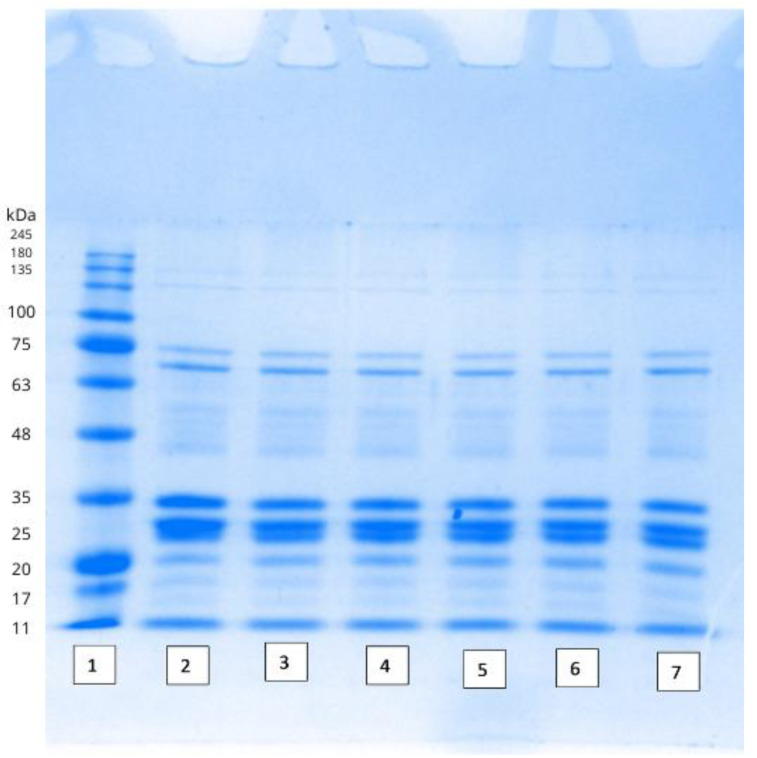
SDS-PAGE gel with the lanes representing the (1) marker, 350 MPa HPP treatment of camel milk, at (2) 0 min, (3) 1 min, (4) 2 min, (5) 3 min, (6) 4 min, and (7) 5 min, respectively.

The presence of psychro-tolerant spoilage causing microorganisms which are not destroyed by initial food processing techniques like pasteurization is an issue that plagues the dairy industry because of the storage temperatures of milk. The growth of the psychro-tolerant species could be partially explained by the initial food processing technique itself. Any technique like HPP or thermal pasteurization could destroy the other protective systems inherent to the preservation of milk like the lactoperoxidase system besides the other microflora. This provides additional nutrient availability and lower barriers to the growth of the psychro-tolerant species. Some studies indicate that HPP has a greater capacity to preserve the “beneficial” components/aspects of milk as opposed to thermal treatments [38,39]. In skimmed milk, it was observed that the lactoperoxidase system was unaffected at pressures ranging from 450 to 700 MPa at 20 °C [39]. Lactoferrin and lactoperoxidase are significant proteins found in camel milk due to their bioactive roles, including antimicrobial and immunomodulatory effects. Based on Figure 2, the molecular weight of lactoferrin corresponds to approximately 80 kDa, while lactoperoxidase aligns with the 78–80 kDa range as supported by previous studies [40]. These proteins are prominently detected, which highlights the unique composition of camel milk in comparison to bovine milk. Lactoferrin’s relatively higher abundance in camel milk is notable for its ability to enhance immune responses and inhibit bacterial growth. The presence of lactoperoxidase further underlines the antimicrobial properties of camel milk, suggesting its suitability as a functional food. However, the lactoferrin content decreased with increasing pressure and treatment time. In another study, the lactoperoxidase system worked synergistically with HPP in reducing *E. coli* populations [41]. However, it did not significantly reduce the population of *Listeria*. Further studies which evaluate the effect of HPP treatment at different time intervals on these systems and the effect this would have on decreasing the spoilage causing microorganisms like TPC, YM, and LAB is needed. It is possible that a milder HPP treatment may have not destroyed the beneficial microflora or decreased the effectiveness of the lactoperoxidase system to that degree, and although the initial microbial counts immediately post the HPP treatment may have been higher, further refrigeration/the presence of the lactoperoxidase or the lactoferrin system may have acted on these semi compromised microorganisms and decreased their populations by a greater number. However, it is not just the spoilage causing microorganisms; the effect of decreasing the HPP treatment in congruence with the activity of the other antimicrobial systems may need to be tested on pathogenic microorganisms too as their presence could prove to be fatal to immunocompromised patients.

### 3.3. Effect of HPP Activity on Proteolytic Profile and Protein Separation in Camel Milk

With respect to protein destruction, the SDS-PAGE analysis was performed to examine the effect of HPP on the proteolytic profile and protein separation in camel milk under varying holding times, and the results are presented in Figure 2.

Results indicated that HPP treatment, even at extended holding times, did not have an effect on the proteolytic activity and casein micelle structure in camel milk. The protein bands of HPP-treated samples displayed similar patterns to those of the control samples, with no significant increase in the number or intensity of bands. The SDS-PAGE analysis (Figure 2) revealed that the most prominent protein bands fall within the 35–48 kDa range, which corresponds to camel milk caseins, particularly β-casein (~37 kDa) and α-casein (~34–36 kDa) [42]. Caseins are the major protein fraction in camel milk, accounting for approximately 52–87% of the total protein content. These proteins contribute significantly to the milk’s nutritional value and digestibility [43].

Although factors like moisture content, pH, salt levels, and processing conditions are known to influence proteolytic activities in milk [44], the treatment of camel milk at 350 MPa for up to 5 min did not significantly alter the protein network. Unlike previous studies where HPP affected protein breakdown in other types of milk, camel milk caseins (α-, β-, and κ-caseins) exhibited resistance to HPP-induced proteolysis, with the proteins largely retaining their molecular weight profiles across all holding times [45,46]. Caseins were primarily discussed because they represent the predominant protein fraction in camel milk. Their molecular characteristics differ from bovine milk caseins, exhibiting a lower degree of phosphorylation and, consequently, reduced stability during processing. As previously noted, camel milk proteins, including caseins, are more vulnerable to denaturation and sedimentation, which can impact their nutritional properties and functional applications. Despite this reduced stability, caseins in camel milk are recognized for their hypoallergenic properties, making camel milk a valuable alternative for individuals allergic to bovine milk proteins [47]. The balance between reduced stability and hypoallergenic potential presents both challenges and opportunities for food applications and nutritional utilization.

The limited effect of HPP on camel milk caseins could be explained by the resilience of casein micelles under pressure, as evidenced by the consistent molecular weight observed across samples. It may also be attributed to the shorter holding time of the HPP treatment that might not have affected the casein micelle structure. No noticeable increase in low-molecular-weight bands was detected, suggesting that extended proteolysis did not occur during HPP treatment. While endogenous proteases such as plasmin are present in camel milk and known to have an affinity for β-caseins, the HPP treatment did not appear to activate or enhance their activity under the conditions applied [15,48].

## 4. Conclusions

The treatment of camel milk with HPP at 350 MPa for 5 min reduced numbers of the tested foodborne pathogens by up to 2 to 3 log cfu/mL and controlled the level of microflora, but after HPP treatment, storage at 4 °C is recommended to facilitate control. HPP treatment inactivates pathogens and inhibits spoilage microorganisms to some extent without the use of heat. Studies incorporating the effect of HPP on the coagulating and aggregation properties of camel milk need to be conducted. Applying HPP technology to camel milk offers significant benefits in regions such as the Middle East, North Africa, and Asia because of its extended shelf life and preserved quality, making it suitable for distribution and international trade. However, the high cost associated with HPP equipment limits its adoption in developing countries, although subsidies or shared facilities could help smaller-scale producers.

## Figures and Tables

**Table 1:** Variations in total plate count population density (log10 (N_0_/N) ± SD cfu/mL) in camel milk samples subjected to 350 MPa HPP for durations of 0, 1, 2, 3, 4, and 5 min, followed by storage periods of 0, 1, 4, 7, and 10 d at temperatures of 4 °C or 10 °C.

Temp	Days	CNL	1 min	2 min	3 min	4 min	5 min	*p*-Value
4 °C	0	0.00 ^abcde^ ± 0.0	−0.45 ^cdef^ ± 0.3	−0.24 ^bcdef^ ± 0.1	−0.25 ^bcdef^ ± 0.5	−0.36 ^bcdef^ ± 0.5	−0.63 ^def^ ± 0.3	0.001
	1	0.42 ^ab^ ± 0.2	−0.61 ^def^ ± 0.4	−0.59 ^def^ ± 0.4	−0.27 ^bcdef^ ± 0.6	−0.26 ^bcdef^ ± 0.4	−0.81 ^f^ ± 0.5	
	4	0.63 ^a^ ± 0.1	0.01 ^abcde^ ± 0.5	−0.02 ^abcde^ ± 0.5	0.13 ^abcd^ ± 0.4	−0.35 ^bcdef^ ± 0.5	−0.71 ^ef^ ± 0.8	
	7	−0.01 ^abcde^ ± 0.3	−0.21 ^bcdef^ ± 0.2	−0.5 ^cdef^ ± 0.2	−0.37 ^cdef^ ± 0.5	−0.15 ^abcdef^ ± 0.6	−0.76 ^ef^ ± 0.5	
	10	0.23 ^abc^ ± 0.5	−0.22 ^bcdef^ ± 0.6	−0.39 ^cdef^ ± 0.4	−0.16 ^bcdef^ ± 0.4	−0.4 ^cdef^ ± 0.6	−0.25 ^bcdef^ ± 0.6	
10 °C	0	0.00 ^hijklm^ ± 0.0	−0.6 ^lm^ ± 0.2	−0.72 ^m^ ± 0.3	−0.48 ^klm^ ± 0.6	−0.45 ^klm^ ± 0.6	−0.63 ^lm^ ± 0.4	0.001
	1	0.76 ^cdefg^ ± 0.1	0.39 ^defghij^ ± 0.4	0.08 ^ghijkl^ ± 0.6	−0.11 ^ijklm^ ± 0.7	−0.26 ^jklm^ ± 0.7	−0.04 ^ijklm^ ± 0.6	
	4	0.85 ^cdef^ ± 0.6	0.18 ^fghijk^ ± 0.7	0.3 ^efghij^ ± 0.4	0.14 ^fghijk^ ± 0.3	0.14 ^fghijk^ ± 0.6	0.15 ^fghijk^ ± 0.4	
	7	1.61 ^ab^ ± 0.5	0.42 ^defghij^ ± 0.5	0.58 ^cdefghi^ ± 0.5	0.69 ^cdefgh^ ± 0.2	0.38 ^defghij^ ± 0.6	0.3 ^efghij^ ± 0.3	
	10	2.17 ^a^ ± 0.4	1.23 ^bc^ ± 0.5	1.1 ^bcd^ ± 0.6	0.96 ^bcde^ ± 0.5	0.73 ^cdefg^ ± 0.7	0.86 ^cdef^ ± 0.4	
4 °C vs. 10 °C	0	NS	0.218	*	0.305	0.688	0.985	
	1	*	*	*	0.525	0.985	*	
	4	0.187	0.453	0.086	0.937	*	*	
	7	*	*	*	*	*	*	
	10	*	*	*	*	*	*	

CNL: Control. ^a–m^: Significant variations (*p* < 0.05) between the means are denoted by distinct letters for each treatment. Initial number (N_0_) of total plate count in camel milk under different temperatures at 4 °C and 10 °C was (4.31 ± 0.69 and 4.12 ± 0.79). * Different letters in each column indicate a statistically meaningful variance (*p* < 0.05) among the averages for identical storage periods at 4 °C and 10 °C temperatures.

**Table 2:** Variations in yeast and mold population density (log10 (N_0_/N) ± SD cfu/mL) in camel milk samples subjected to 350 MPa HPP for durations of 0, 1, 2, 3, 4, and 5 min, followed by storage periods of 0, 1, 4, 7, and 10 d at temperatures of 4 °C or 10 °C.

Temp	Days	CNL	1 min	2 min	3 min	4 min	5 min	*p*-Value
4 °C	0	0.00 ^bcd^ ± 0.0	−0.48 ^bcde^ ± 0.3	−0.22 ^bcde^ ± 0.2	−0.14 ^bcd^ ± 0.5	−0.29 ^bcde^ ± 0.4	−0.65 ^de^ ± 0.3	0.001
	1	−0.05 ^bcd^ ± 0.3	−0.61 ^cde^ ± 0.3	−0.63 ^de^ ± 0.4	−0.40 ^bcde^ ± 0.5	−0.38 ^bcde^ ± 0.4	−0.95 ^e^ ± 0.4	
	4	0.30 ^ab^ ± 0.1	−0.40 ^bcde^ ± 0.3	−0.29 ^bcde^ ± 0.3	−0.02 ^bcd^ ± 0.3	0.05 ^bcd^ ± 0.3	−0.54 ^cde^ ± 0.3	
	7	0.17 ^bc^ ± 0.4	−0.53 ^cde^ ± 0.4	−0.31 ^bcde^ ± 0.2	−0.31 ^bcde^ ± 0.2	0.10 ^bcd^ ± 0.3	−0.51 ^cde^ ± 0.4	
	10	1.00 ^a^ ± 0.7	0.08 ^bcd^ ± 0.5	−0.15 ^bcde^ ± 0.5	0.01 ^bcd^ ± 0.6	−0.08 ^bcd^ ± 0.7	0.04 ^bcd^ ± 0.7	
10 °C	0	0.00 ^defghi^ ± 0.0	−1.14 ^kl^ ± 0.4	−1.05 ^jkl^ ± 0.4	−0.64 ^hijkl^ ± 0.9	−0.83 ^ijkl^ ± 0.7	−1.17 ^l^ ± 0.5	0.001
	1	0.17 ^bcdefghi^ ± 0.5	−0.38 ^efghijkl^ ± 0.3	−0.62 ^ghijkl^ ± 0.4	−0.5 ^fghijkl^ ± 0.4	−0.7 ^ijkl^ ± 0.5	−0.82 ^ijkl^ ± 0.7	
	4	0.58 ^abcde^ ± 0.7	−0.01 ^defghi^ ± 0.7	0.03 ^defghi^ ± 0.4	−0.04 ^defghij^ ± 0.3	−0.15 ^defghijk^ ± 0.8	−0.09 ^defghij^ ± 0.3	
	7	1.06 ^abc^ ± 0.7	0.65 ^abcd^ ± 1.1	0.34 ^bcdefgh^ ± 0.8	0.39 ^bcdefg^ ± 0.9	0.09 ^cdefghi^ ± 0.7	−0.13 ^defghijk^ ± 0.4	
	10	1.56 ^a^ ± 1.0	1.18 ^ab^ ± 0.8	0.61 ^abcde^ ± 0.7	0.45 ^bcdef^ ± 0.7	0.37 ^bcdefgh^ ± 0.7	0.48 ^bcdef^ ± 0.7	
4 °C vs. 10 °C	0	NS	0.101	*	0.145	0.053	*	
	1	0.181	0.216	0.948	0.661	0.084	0.597	
	4	0.327	0.227	0.108	0.859	0.482	*	
	7	*	*	*	*	0.951	0.058	
	10	0.175	*	*	0.102	0.107	0.156	

CNL: Control. ^a–l^: Significant variations (*p* < 0.05) between the means are denoted by distinct letters for each treatment. Initial number (N_0_) of total plate count in camel milk under different temperatures at 4 °C and 10 °C was (4.22 ± 0.84 and 4.52 ± 1.02). * Different letters in each column indicate a statistically meaningful variance (*p* < 0.05) among the averages for identical storage periods at 4 °C and 10 °C temperatures.

**Table 3:** Variations in lactic acid bacterial population density (log10 (N_0_/N) ± SD cfu/mL) in camel milk samples subjected to 350 MPa HPP for durations of 0, 1, 2, 3, 4, and 5 min, followed by storage periods of 0, 1, 4, 7, and 10 d at temperatures of 4 °C or 10 °C.

Temp	Days	CNL	1 min	2 min	3 min	4 min	5 min	*p*-Value
4 °C	0	0.00 ^abcd^ ± 0.0	−0.09 ^abcd^ ± 0.3	0.14 ^abc^ ± 0.2	0.1 ^abc^ ± 0.5	−0.15 ^abcd^ ± 0.3	−0.19 ^abcd^ ± 0.3	0.001
	1	0.13 ^abc^ ± 0.5	−0.36 ^abcd^ ± 0.3	−0.44 ^bcd^ ± 0.4	−0.12 ^abcd^ ± 0.5	0.01 ^abcd^ ± 0.4	−0.56 ^cd^ ± 0.5	
	4	0.12 ^abc^ ± 1.2	−0.31 ^abcd^ ± 0.6	−0.27 ^abcd^ ± 0.9	−0.05 ^abcd^ ± 0.7	0.43 ^ab^ ± 0.3	−0.91 ^d^ ± 0.9	
	7	0.35 ^abc^ ± 0.6	0.03 ^abcd^ ± 0.1	0.1 ^abc^ ± 0.4	0.31 ^abc^ ± 0.3	0.42 ^ab^ ± 0.4	−0.21 ^abcd^ ± 0.4	
	10	0.38 ^abc^ ± 0.5	0.01 ^abcd^ ± 0.5	−0.22 ^abcd^ ± 0.4	0.21 ^abc^ ± 0.4	0.38 ^abc^ ± 0.7	0.54 ^a^ ± 0.5	
10 °C	0	0.00 ^g^ ± 0.0	−0.08 ^g^ ± 0.2	−0.02 ^g^ ± 0.3	0.37 ^fg^ ± 0.7	0.28 ^fg^ ± 0.7	−0.04 ^g^ ± 0.3	0.001
	1	0.55 ^cdefg^ ± 0.3	0.56 ^cdefg^ ± 0.4	0.25 ^fg^ ± 0.4	0.41 ^efg^ ± 0.4	0.29 ^fg^ ± 0.7	0.24 ^fg^ ± 0.7	
	4	0.7 ^bcdefg^ ± 1.0	0.67 ^bcdefg^ ± 0.5	0.53 ^defg^ ± 0.7	0.55 ^cdefg^ ± 0.5	0.74 ^bcdefg^ ± 0.6	0.33 ^fg^ ± 1.1	
	7	1.51 ^ab^ ± 0.4	1.47 ^abc^ ± 0.4	1.46 ^abc^ ± 0.4	1.34 ^abcd^ ± 0.1	1.32 ^abcde^ ± 0.2	1.12 ^abcdef^ ± 0.1	
	10	1.39 ^abcd^ ± 0.2	1.8 ^a^ ± 0.4	1.6 ^ab^ ± 0.5	2.02 ^a^ ± 1.0	1.56 ^ab^ ± 0.6	1.41 ^abcd^ ± 0.6	
4 °C vs. 10 °C	0	NS	0.939	0.238	0.407	0.227	0.369	
	1	*	*	*	*	0.229	*	
	4	0.212	*	0.023	*	0.127	*	
	7	*	*	*	*	*	*	
	10	*	*	*	*	*	*	

CNL: Control. ^a–g^: Significant variations (*p* < 0.05) between the means are denoted by distinct letters for each treatment. Initial number (N_0_) of total plate count in camel milk under different temperatures at 4 °C and 10 °C was (3.93 ± 0.41 and 3.52 ± 0.29). * Different letters in each column indicate a statistically meaningful variance (*p* < 0.05) among the averages for identical storage periods at 4 °C and 10 °C temperatures.

## Data Availability

The original contributions presented in the study are included in the article, further inquiries can be directed to the corresponding author.
